# Automated deep-learning quantification of intramuscular fat in lumbar spine muscles on Dixon MRI: validation and normative reference values from 173 healthy adults

**DOI:** 10.1186/s12880-026-02329-9

**Published:** 2026-03-31

**Authors:** Germán Balerdi, Johann Henckel, Anna Di Laura, Alister J. Hart, Martín A. Belzunce

**Affiliations:** 1 Centro Universitario de Imágenes Médicas (CEUNIM), Escuela de Ciencia y Tecnología, Universidad Nacional de San Martín, San Martín, Buenos Aires Argentina; 2 Royal National Orthopaedic Hospital, Stanmore, UK; 3 Institute of Orthopaedics and Musculoskeletal Science, University College London, Stanmore, UK; 4 Instituto de Ciencias Físicas (ICIFI UNSAM-CONICET), Escuela de Ciencia y Tecnología, Universidad Nacional de San Martín, San Martín, Buenos Aires Argentina; 5 Consejo Nacional de Investigaciones Científicas y Tecnológicas (CONICET), Buenos Aires, Argentina; 6 Cleveland Clinic London, London, UK

**Keywords:** MRI, Lumbar spine, Muscle health, Deep Learning, Intramuscular fat, Musculoskeletal, Quantitative MRI

## Abstract

**Background:**

Intramuscular fat (IMF) accumulation in lumbar‑spine muscles is associated with sarcopenia, low‑back pain and poorer surgical outcomes. Dixon MRI allows the measurement of IMF with voxel-wise fat fraction (FF) mapping, but quantitative use in research or clinics is limited by labour-intensive 3D muscle segmentation and by the absence of healthy reference data. We present a lightweight pipeline that requires a small number of labelled images yet delivers accurate segmentation and Dixon-based reference ranges for lumbar spine IMF in asymptomatic adults with diverse physical activity levels.

**Methods:**

Twenty-six Dixon scans (44.8 ± 14.3 years old; 12 males / 14 females) were manually annotated for psoas, iliacus, quadratus lumborum and the erector spinae + multifidus complex. A 3D U‑Net was trained with a realistic data augmentation scheme that combines small rigid rotations and non-linear B-spline deformations mimicking real anatomical variability, enhancing generalisation. Mean fat fraction (FF) of each muscle was extracted from Dixon FF images. Segmentation (Dice, relative volume difference, Hausdorff distance) and FF estimation accuracy was assessed with a five‑fold Monte‑Carlo cross‑validation and was compared to a multi-atlas approach. FF agreement with manual measurements was examined using linear regression and Bland–Altman analysis. The trained model then processed the complete study cohort of 173 healthy adults (20–70 years old; sedentary to highly active) to derive healthy subjects FF percentiles.

**Results:**

Automated FF strongly correlated with manual derived values (R² = 0.96), showing no systematic bias. The 3D U-Net achieved Dice coefficients of up to 0.936 (erector spinae + multifidus) and demonstrated superior performance over multi-atlas segmentation across all metrics (*p* < 0.01). The model was also able to capture different characteristic FF values for each muscle, with median (IQR) normative FF values of 21.8% (17.4–26.9%) for erector spinae + multifidus, 18.0% (14.9–22.2%) for quadratus lumborum, 13.4% (10.5–16.3%) for psoas and 11.6% (9.5–13.8%) for iliacus, with significant sex and activity differences (*p* < 0.01).

**Conclusion:**

With fewer than 30 manual annotations, a 3D U-Net model enhanced by anatomically realistic data augmentation, provides a reliable and accurate solution for automated IMF quantification from Dixon MRI. We deliver novel Dixon MRI reference values of lumbar spine muscle composition in healthy adults. Both the automated estimation of FF, and the reference FF values reported here, facilitates the study of sarcopenia, spinal pathologies, and muscle degeneration.

**Supplementary Information:**

The online version contains supplementary material available at 10.1186/s12880-026-02329-9.

## Background

Fat infiltration and atrophy of the lumbar spine muscles are associated with sarcopenia and many spinal degenerative conditions, contributing to pain, functional limitation and poorer surgical outcomes [1–6]. Accumulation of intramuscular fat (IMF) (that is, fat within the muscle fascia) reduces muscle contractile capacity, as muscle fibres are replaced by non-contractile tissue [7–9], making quantitative IMF a clinically relevant biomarker. Accurate assessment tools are needed to aid diagnosis, treatment, and monitoring of muscle health [10–17].

Magnetic‑resonance imaging (MRI) is the reference standard for muscle evaluation. Dixon MRI is a quantitative sequence that enables the measurement of proton-density fat fraction (FF) that sensitively capture IMF once individual muscles are accurately segmented [18–22]. The use of quantitative MRI to study muscle health has grown steadily in recent years, both to study myopathies [23–25], as well as sarcopenia and muscle health in healthy subjects with different levels of physical activity [17, 21, 22, 26–29]. The major obstacle is that 3D, muscle‑specific segmentation remains a labour‑intensive manual task, limiting both clinical adoption and large‑cohort research.

Deep learning, particularly convolutional neural networks (CNNs), such as U-Nets [30], has revolutionised medical image segmentation, achieving high accuracy in various applications [31]. Previous studies have demonstrated its effectiveness in segmenting musculoskeletal structures, including the psoas and iliacus muscles [14, 32–37]. However, the lack of large, annotated datasets remains a challenge in training robust deep learning models for muscle segmentation. Due to this, realistic and domain specific data augmentation techniques are required [38, 39]. In addition, almost no studies report normative lumbar‑spine FF ranges in healthy adults, even though such baselines are essential for stratifying disease severity and monitoring interventions.

This study proposes a novel approach that combines a 3D U-Net with deformation-based data augmentation techniques, needing only 26 manual annotations, to automatically quantify the fat infiltration of the lumbar spine muscles from Dixon MRI. After evaluating its performance with a 5-fold Monte‑Carlo cross‑validation, and comparing it with a multi‑atlas baseline, we deploy the network on 173 asymptomatic adults with different levels of physical activity and provide reference FF values for the psoas, iliacus, quadratus‑lumborum and erector‑spinae + multifidus muscles.

## Materials and methods

We implemented and evaluated a deep-learning method for fully automated 3D quantification of IMF in the lumbar spine muscles, trained with 26 3D Dixon MRI images from a study looking at muscle health and physical activity. It automatically segments the psoas (P), iliacus (I), quadratus lumborum (QL), and erector spinae and multifidus combined (ES + M) from in-phase Dixon MRI and computes the IMF in each muscle as the mean FF value for each muscle from the Dixon FF image. The method was then applied to the full study cohort to obtain reference IMF values for this muscle group.

### Study cohort

We analysed 173 Dixon MRI examinations acquired for a study of muscle health and physical activity. The study comprised 87 sedentary volunteers and 86 recreational cyclists who underwent axial Dixon MRI. Overall, 92 participants were female and 81 male, with a mean (SD) age of 43.5 (12.8) years and BMI of 25.7 (5.6) kg/m^2^.

The images were acquired in a 3T scanner (Siemens Magneton Vida, Erlangen, Germany) using a body coil and a standardised protocol that includes a Dixon sequence. The scanning protocol consisted of an axial TSE Dixon sequence (slice thickness 1.5 mm, spacing between slices 1.95 mm, repetition time (TR) 4570 ms, echo time (TE) 45 ms, number of excitations 1, number of echoes 14, flip angle 120°), with a field of view (FOV) that covered axially from 1 cm below the lesser trochanter to the origin of the psoas muscle at the level of the L1 vertebra. The voxel size was 0.47 × 0.47 × 1.95 mm3.

### Manual ground truth

**Fig. 1:**
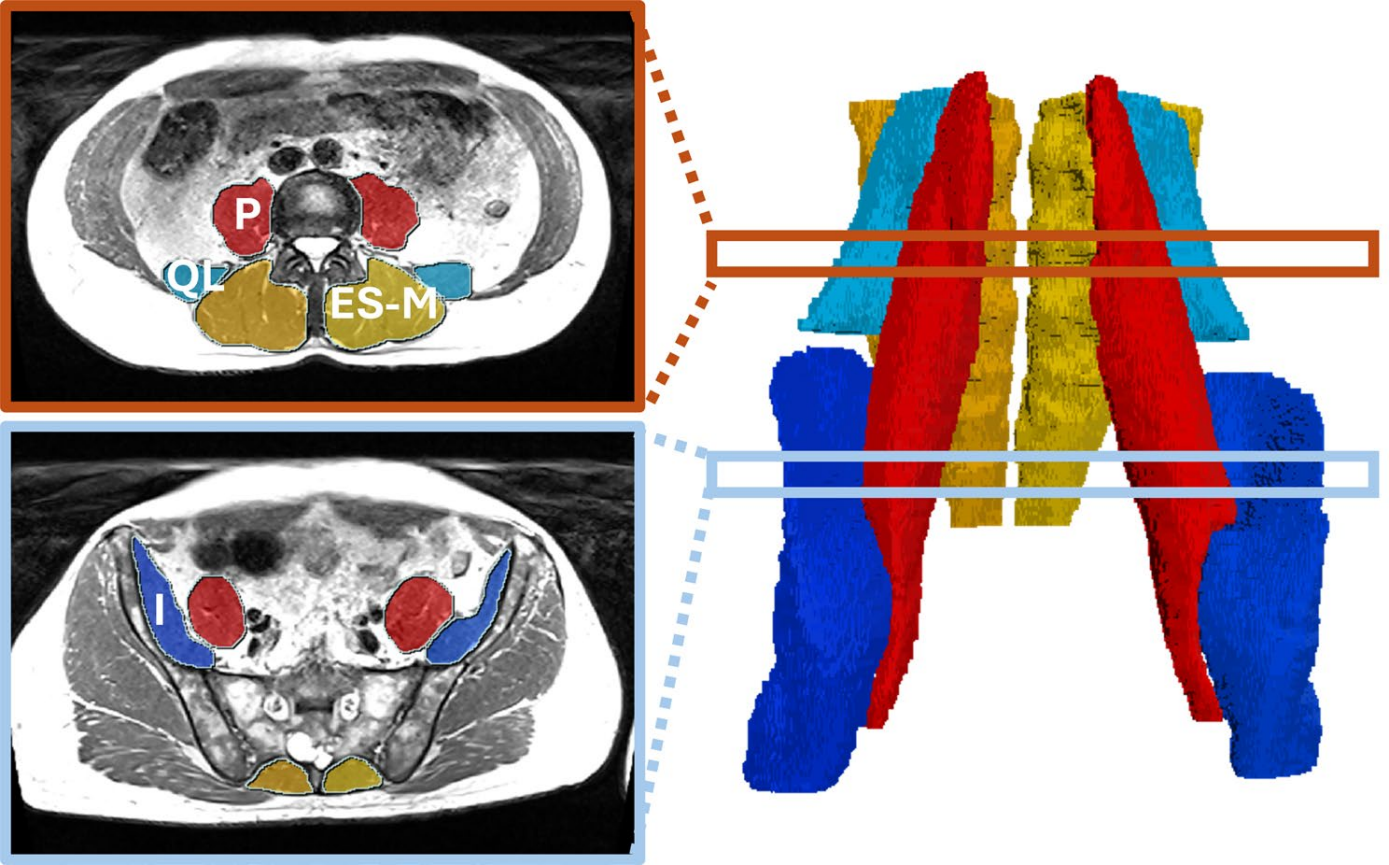
Example of a manual segmentation. On the right side, a 3D model of the manually segmented psoas (P), iliacus (I), quadratus lumborum (QL), erector spinae and multifidus muscles (ES + M). On the left, axial slices of the in-phase Dixon MRI with colour masks of the segmentations at two different levels of the spine

A subset of 26 images was manually segmented by an expert operator using Simpleware ScanIP (Version 2024; Synopsys, Inc., Mountain View, USA). Segmented muscles were P, I, QL, and ES + M. The muscle masks included the intramuscular fat located within the fascia compartment. A special case to consider is the posterior region of the ES + M complex, where an epimuscular fat tent is often observed in subjects with fatty atrophy. In our segmentations, we followed the method described in [40], using the epimyseal border to delineate the posterior boundary, thereby excluding the epimuscular fat [41, 42]. Figure 1 shows an example of manual segmentation, and Supplementary Fig. 1 illustrates the exclusion of the epimuscular fat. The twenty-six MRI scans correspond to 12 female and 14 male individuals with a mean (SD) age of 44.8 (14.3) years and a BMI of 26.8 (5.9) kg/m^2^. Seventeen of them were part of the sedentary group and nine were cyclists.

### Pre-processing and data augmentation

**Fig. 2:**
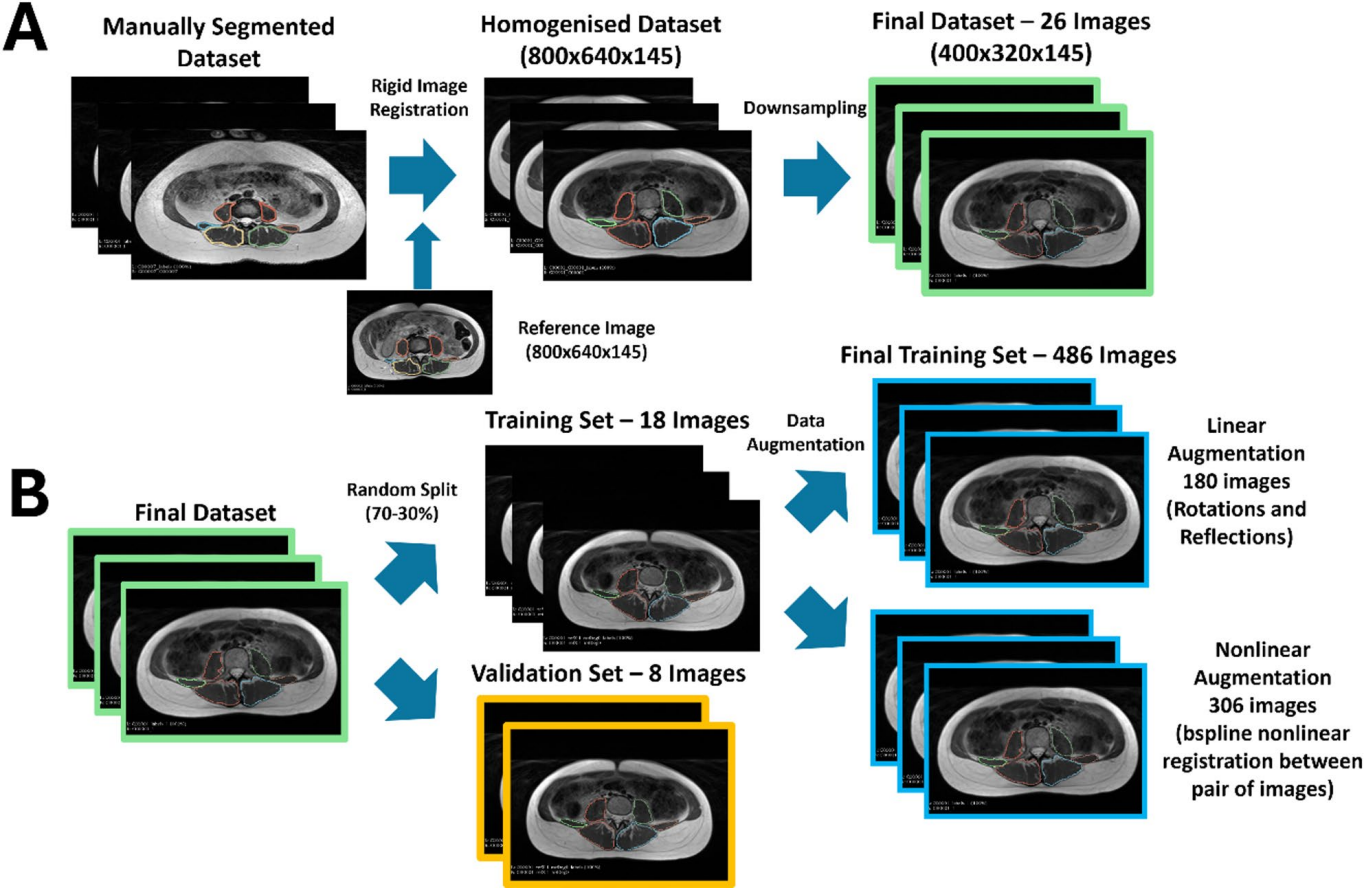
Data homogenisation (**A**) and augmentation process (**B**). A homogeneous dataset in terms of voxel size and matrix dimensions was created from the 26 manually segmented 3D in-phase Dixon images (**A**). This data set was then randomly split into a training and validation set for each fold of the cross-validation, and the training images were augmented using linear and nonlinear techniques to obtain a training set of 486 images. No validation images were used in training, either directly or indirectly

All images were pre-processed to enhance the consistency of the input data. In this process, all images were rigid registered to a reference image with Simple Elastix using the normalised cross correlation as similarity metric [43]. The homogenised images were then downsampled to half the size in the axial plane to reduce the memory requirements of the model, which translated into a four-fold reduction in its memory size with respect to the original images, without a significant loss in image quality. As a result, all the images had voxel sizes of 0.94 × 0.94 × 1.95 mm^3^, a matrix size of 400 × 320 × 145 voxels and had the same geometrical space. This process is depicted in Fig. 2-A.

To improve model generalisation, we applied data augmentation techniques to artificially increase the size of the training dataset. In addition to linear augmentation, here we propose a nonlinear deformation-based augmentation that accounts for the large anatomical variability in the abdominal and pelvic regions (Fig. 2-B):

- Linear Augmentation: Clockwise and anticlockwise rotations of 5° and 10° were applied to the original and flipped images on the x axis (medial-lateral) to account for asymmetries and variability in the patient positioning.
- Nonlinear Augmentation: We generated deformed images by registering pairs of original images using B-spline nonlinear transformations to create realistic deformations that simulate anatomical variability. Nonrigid B-spline registration was implemented using SimpleElastix [43] with normalized cross-correlation (NCC) as similarity metric and the adaptive stochastic gradient descent algorithm with 500 iterations and 2048 samples. The number of iterations was selected to stop early in the registration process, creating virtual anatomies between the two images being registered. Supplementary Fig. 2 shows two examples of this data augmentation process.

The data augmentation process was applied only to the training set of each of the trained models.

### 3D U-Net architecture

We implemented a CNN with a 3D U-Net architecture. The network consists of an encoder-decoder structure with skip connections, as proposed by Ronneberger et al. [30]. The input to the model was the pre-processed 3D MRI Dixon in-phase images, and the output was a multi-label binary mask with hot encoding representing the segmentation of each muscle. The architecture consisted of 4 encoder and 4 decoder layers, with 16 convolutional filters in the first layer. It had a total of 2.5 million parameters.

### Trained models

The 3D U-Net was evaluated using a five-iteration fixed-split Monte Carlo cross validation. In this method, the training and validation sets were randomly sampled five times. For each iteration, 18 3D images were used for training (approximately 70% of the data) and the remaining 8 were used for validation (approximately 30% of the data). This process was repeated five times, ensuring that each 3D image appeared once in the validation set.

Data augmentation was applied only to the training subsets, generating a total of 486 augmented 3D images per fold (180 linearly and 306 nonlinearly augmented), as described previously. The models’ performance was evaluated on the validation set of each fold, and the results were averaged across the five folds to ensure robust evaluation of the segmentation method. All training and validation were performed on full 3D in-phase Dixon images. No validation image was used in training, either directly or indirectly through non-linear augmentation.

The five cross-validation models were trained using binary cross entropy as loss function and the ADAM optimiser with a learning rate of 0.0001. The model was trained with early stopping to obtain the minimum loss for the validation set.

### Baseline: Multi-atlas segmentation

We implemented a multi-atlas segmentation method as a baseline for comparison, as this type of methods works well with low numbers of manually segmented images. The multi-atlas approach was similar to our previous work for the gluteal muscles [44, 45]. An 18‑atlas library (subset of the 26 labelled scans) was used in a leave‑one‑out scheme. To segment an image, every atlas in the library is registered to the target image to be segmented, which are then sorted in descending similarity order using the NCC as metric. The labels of the 5 most similar atlases are propagated to the target image space and subsequently fused into a single label for each muscle using majority voting [46].

For image registration, an affine followed by a non-rigid *B-spline* registration [47] was implemented using SimpleElastix [43, 48]. NCC was used as a similarity metric and the cost function was minimised using the adaptive stochastic gradient descent algorithm [49] with 2000 iterations and 2048 samples. A pyramidal scheme of four layers with downsampling factors of 8, 4, 2 and 1 was employed to improve the registration. These parameters have previously been optimized in terms of segmentation performance.

All multi-atlas segmentation labels were post-processed to improve the spatial consistency of the labels. Post-processing consisted of applying a soft-tissue intensity mask to remove subcutaneous fat voxels from the labels and then a applying morphological filter to fill holes in the labels.

### Fat fraction measurement

We created FF images for each of the 3D scans of the dataset, which is the ratio image between the fat image and the sum of the water and fat Dixon images. For each muscle, the mean FF was then calculated by averaging the voxel values within the corresponding 3D muscle mask. All measurements were performed across the entire muscle volume, ensuring a comprehensive quantification of fat infiltration rather than relying on individual slices or regions.

### Evaluation metrics

Segmentation performance was evaluated using three complementary metrics: (a) the dice similarity coefficient (Dice) to measure the overlap between the predicted segmentation and the ground truth. This metric is the most widely used overall metric for assessing segmentation performance, especially relevant for evaluating agreement in both volume and shape; (b) the relative volume difference (RVD) that assesses the accuracy of the volume estimation, calculated as the percentage difference in total volume between the predicted and ground-truth segmentations; and (c) the Hausdorff distance (HD), which measures the maximum surface-to-surface distance between the predicted and manual segmentation boundaries, capturing the worst-case discrepancy at the borders and being particularly useful for assessing the accuracy of shape delineation. These metrics were also computed for the multi-atlas method.

Each metric offers complementary information: Dice evaluates overall overlap, RVD focuses on volumetric agreement, and HD emphasizes shape and surface accuracy. Note that Dice and RVD can be lower for smaller muscles due to higher relative sensitivity to minor boundary mismatches.

To assess the accuracy of the fat fraction measurements, we used a Bland-Altman plot to detect systematic errors and fit a linear regression to automated versus manual scatter plots to assess the strength of the relationship. Both Bland-Altman and linear regressions were performed for the validation set of all cross-validation folds together, including repeated images in the validation set of different models.

Metrics were computed on full masks and on masks cropped to the manually annotated axial extent to compensate for limited psoas coverage in some scans. This ensured fair and consistent comparisons between automated and ground truth measurements across all subjects.

### Statistical analysis

The mean (SD) was computed across folds for all the evaluation metrics. The median (IQR) was computed for the evaluation metrics of each fold of the 3D U-Net and the multi-atlas segmentations. For statistical comparison between the 3D U-Net and the multi-atlas method, we used a Kruskal-Wallis tests, with a significance level of *p* < 0.05.

For the fat infiltration measurements, we computed the median (IQR) for the average of left and right FF values of each muscle. We compared the distribution of FF values between the manual and deep learning-based measurements for each muscle, using Kruskal-Wallis tests.

In the full cohort, Type III ANCOVA assessed the contribution of age, sex, BMI and activity group to IMF. Partial eta squared (η²ₚ) were used to quantify the effects sizes of each covariate.

## Results

### Segmentation accuracy

Deep learning 3D U-Net segmentation achieved excellent performance in terms of segmentation accuracy when evaluated with a 5-random sample cross validation, which ensures robust assessment across the entire dataset. Table 1 summarises the average metrics for Dice, RVD, and HD across all models for the validation set, for each muscle group.

**Table 1:** Mean (SD) segmentation accuracy metrics across folds for the 3D U-Net cross-validation models

	Left Side				Right Side			
	*P*	l	QL	ES + M	*P*	l	QL	ES + M
Dice	0.927 (0.004)	0.897 (0.009)	0.871 (0.007)	0.936 (0.007)	0.923 (0.006)	0.885 (0.012)	0.857 (0.015)	0.936 (0.004)
RVD [%]	-2.6 (2.5)	2.1 (6.4)	-2.1 (5.5)	-0.8 (2.9)	0.2 (3.6)	0.4 (10.1)	1.2 (8.0)	-1.5 (2.3)
Hausdorff Distance	7.7 (0.9)	7.7 (1.0)	5.4 (0.5)	8.2 (0.7)	8.8 (1.4)	7.4 (1.3)	5.8 (0.7)	8.5 (1.8)

The mean (SD) Dice score across folds was 0.904 for all muscle groups and side together, with the highest performance observed for the complex ES-M with 0.936 (0.005) and the lowest for the QL with 0.864 (0.011). In terms of RVD, the mean (SD) ranged from − 2.6% (2.5%) for the left psoas to 2.1% (6.4%) for the left iliacus, demonstrating reliable volume estimation. Because segmentation errors occurred primarily where psoas and iliacus merge, we also evaluated the composite iliopsoas. Dice and RVD improved to 0.920 (0.004) and 0.4% (4.9%), respectively.

The HD was lowest for QL, being 5–9 mm for all muscles, indicating accurate boundary delineation. The lower HD but simultaneously lower Dice were due to the small size of QL. No significant differences were found in performance between sides.

### Comparison with baseline

**Fig. 3:**
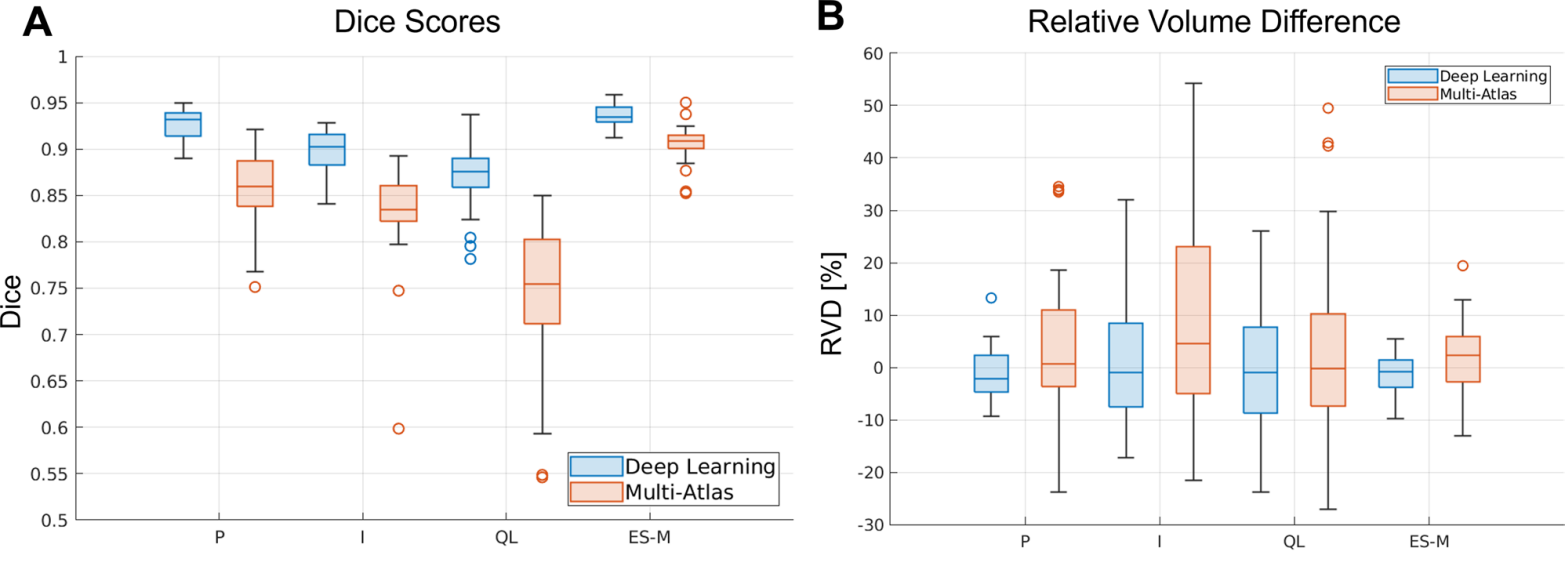
Boxplots of Dice scores (**A**) and RVD values (**B**) for each muscle, for the deep learning (blue) and the multi-atlas (red) methods. For the deep learning method, the segmentations for the validation set of the five cross validation folds were included. On each box, the central mark is the median, the edges of the box are the 25th and 75th percentiles. The outliers are plotted individually

The 3D U‑Net outperformed the multi‑atlas method on every metric (all *p* < 0.001). Figure 3 displays Dice (A) and RVD (B) distributions. The deep‑learning boxes pool 40 validation segmentations (8 scans × 5 folds), while multi‑atlas results are based on the 26 manually labelled scans.

**Fig. 4:**
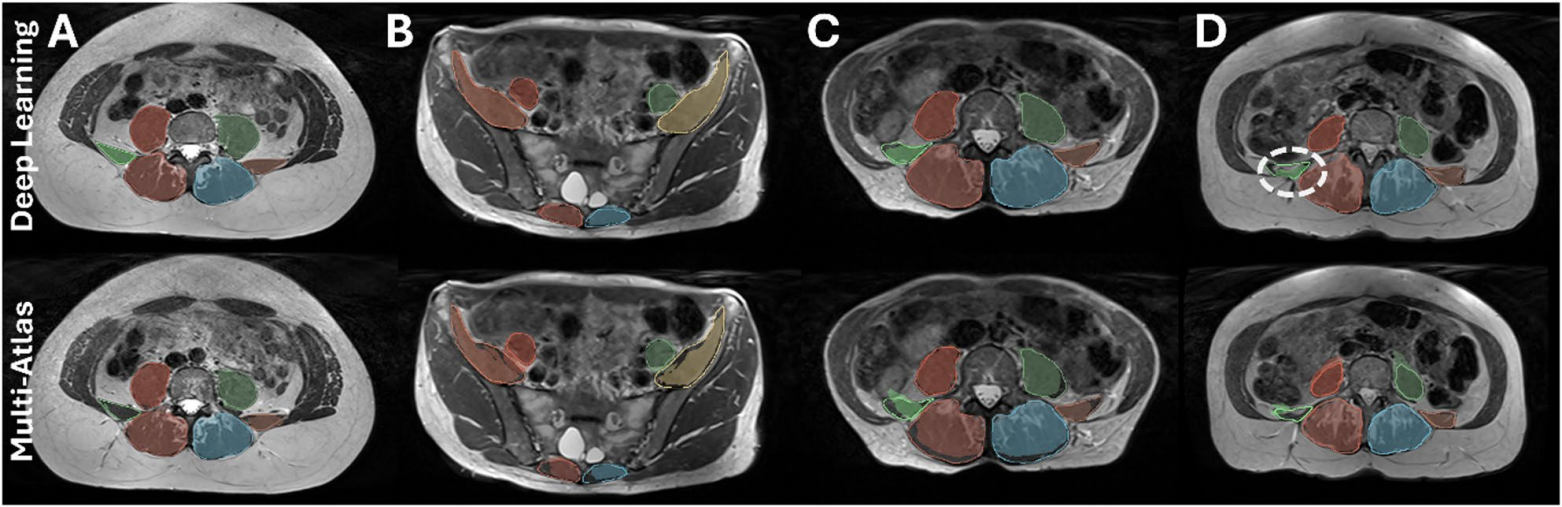
Comparison of automated muscle segmentation results obtained with the deep learning (top row) and multi-atlas methods (bottom row) for three subjects with varying BMIs and degrees of fat infiltration (**B** and **C** correspond to the same subject). Automated segmentations are represented by shaded regions, while the reference manual segmentations are indicated by outlines on each image. In panel **D**, the segmentation of the quadratus lumborum (QL) muscle is highlighted, illustrating the case with the lowest accuracy due to full fatty atrophy of this muscle. The mean fat fraction (FF) values from manual, deep learning and multi-atlas segmentations, respectively, were: **A**) *P* = 14.5%, 14.2%, 19.8%; QL = 10.6%, 10.1%, 20.8%, ES + M = 21.8%, 21.6%, 27.3%; **B**-**C**) *P* = 12.7%, 12.2%, 16.5%; QL = 11.6%, 11.7%, 24.7%, ES + M = 25.7%, 25.0%, 29.1%; **D**) *P* = 14.3%, 14.6%, 25.7%; QL = 22.7%, 18.5%, 34.2%, ES + M = 45.7%, 43.8%, 40.5%. All FF values correspond to the full 3D volume of each segmented muscle

Figure 4 visually compares the segmentation performance of both methods in four representative subjects with varying levels of BMI and degrees of muscle fat infiltration. The deep learning method demonstrates superior capability in accurately segmenting regions where the multi-atlas approach struggled, primarily due to the significant anatomical variability observed in the lumbar spine muscles. Additionally, Fig. 4-D specifically highlights the case with the lowest segmentation accuracy for the deep learning method. This instance, which belongs to the outliers for the quadratus lumborum (QL) muscle in Fig. 3, clearly illustrates complete fatty infiltration in the subject’s right QL muscle, which poses challenges for accurate automated segmentation.

### Fat fraction measurement accuracy

**Fig. 5:**
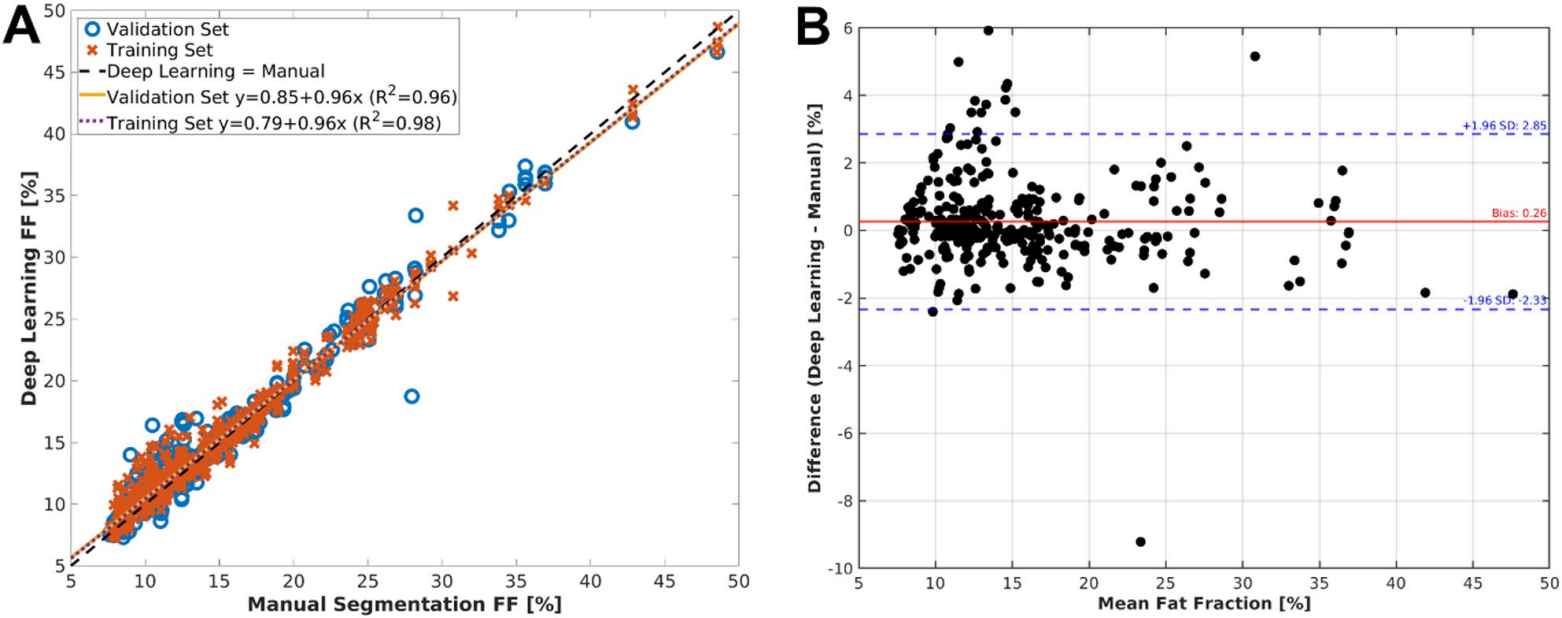
(**A**) Scatter plot comparing fat fraction (FF) values obtained from deep learning versus manually segmented images for all lumbar spine muscles. Each point represents the mean FF across the entire 3D muscle volume for one muscle in one subject. Data points from both the training and validation sets are included, with distinct markers representing each muscle. The dashed line indicates perfect agreement (automated = manual), while solid lines depict linear regression fits separately for training and validation datasets. (**B**) Bland-Altman analysis of the FF of all muscles, comparing deep learning and manual segmentations. The solid lines represent mean differences (bias), and dashed lines indicate limits of agreement (± 1.96 standard deviations). Each plot shows the mean FF of the two methods on the x-axis against their differences on the y-axis, illustrating consistency and variability across methods

Automated and manual FF values were highly correlated with R² = 0.98 for the training set, and R² = 0.96 for the validation set, demonstrating excellent agreement and indicating robust generalisation capability of the deep learning models. Figure 5-A shows a scatter plot comparing FF values derived from automated deep learning segmentation and manual segmentation in all lumbar spine muscles. The Bland–Altman analysis (Fig. 5-B) showed negligible bias (0.1%) and narrow 95% limits of agreement (–2.0% to + 2.4%). Per‑muscle Bland–Altman plots (Supplementary Fig. 3) confirmed excellent agreement for psoas and ES + M, with slightly wider limits for iliacus and QL, and a single QL outlier (muscle with fatty atrophy in Fig. 4-D). No systematic bias was observed for any muscle group, reinforcing the reliability of the deep learning approach.

Moreover, no statistically significant differences were found for the FF distributions between automated and manual measurements for any of the muscle groups (*p* = 0.77 for P, *p* = 0.23 for I, *p* = 0.46 for QL, and *p* = 0.74 for ES + M). The median (IQR) FF values obtained from deep learning were 15.0% (12.9–16.5%) for P, 10.6% (8.8–12.7%) for I, 12.0% (10.4–13.6%) for QL, and 24.1% (19.0–27.6%) for ES + M; the corresponding values obtained from manual segmentation were 15.6% (12.9–16.1%), 10.0% (8.8–11.7%), 11.6% (10.6–13.3%) and 23.7% (19.0–26.6%), respectively.

### Fat fraction reference values and determinants in healthy adults

**Fig. 6:**
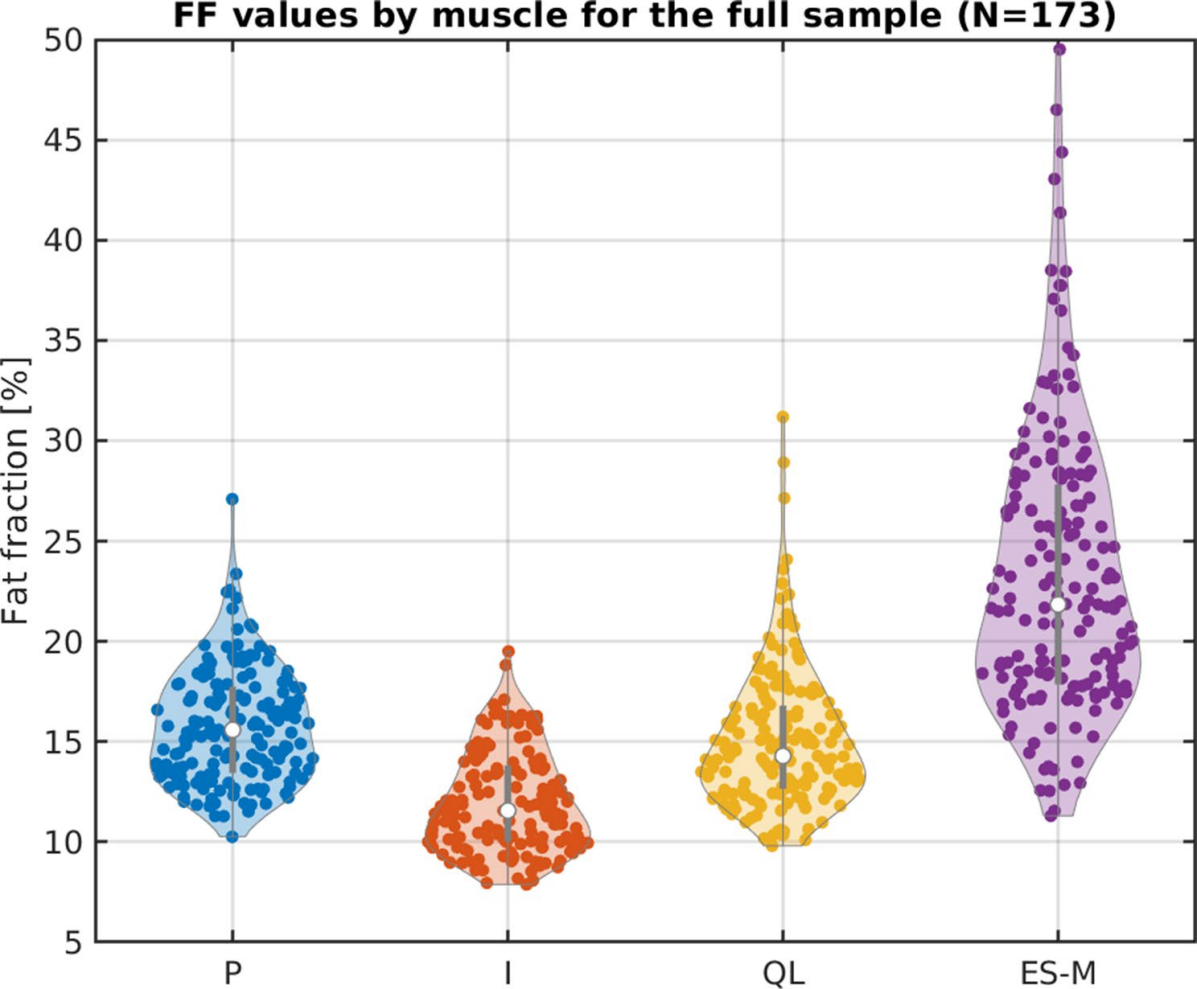
Violin plots illustrating the distribution of FF values for the full dataset compromising sedentary and physically active subjects measured with the deep learning-based automated method

Using the trained U‑Net on the entire cohort (*N* = 173), the median (IQR) FF values were 15.6% (13.5–17.7%) for P, 11.6% (10.0–13.8%) for I, 14.3% (12.7–16.8%) for QL, and 21.8% (17.9–27.8%) for ES + M respectively, showing distinct FF distributions across muscles, illustrated in Fig. 6 with violin plots.

ANCOVA analysis revealed that age had the largest effect size on FF for QL (Partial η²=0.40) and ES + M (Partial η²=0.34), while being in the sedentary or physically active groups for P (Partial η²=0.40) and I (Partial η²=0.45). Sex was a significant contributor for FF for I, QL and ES + M, but with an intermediate effect. Collectively, these findings suggest that age and physical activity substantially contribute to the variance of the lumbar spine fat fraction, whereas BMI and sex appear less influential in this model. Table 2 reports the full results for the ANCOVA analysis for each muscle.

**Table 2:** Results of the Type III ANCOVA analysis examining determinants of intramuscular fat fraction for each muscle

Muscle	Significant Covariates	F (df, 166)	*p*	Partial η²
Psoas	Group	37.31 (3, 166)	*p* < 0.01	0.403
	Age (years)	24.96 (1, 166)	*p* < 0.01	0.131
	BMI	23.49 (1, 166)	*p* < 0.01	0.124
Iliacus	Group	45.47 (3, 166)	*p* < 0.01	0.451
	Age (years)	37.63 (1, 166)	*p* < 0.01	0.185
	Sex	21.10 (1, 166)	*p* < 0.01	0.113
	BMI	47.17 (1, 166)	*p* < 0.01	0.221
Quadratus Lumborum	Group	16.63 (3, 166)	*p* < 0.01	0.231
	Age (years)	110.47 (1, 166)	*p* < 0.01	0.400
	Sex	20.55 (1, 166)	*p* < 0.01	0.110
	BMI	21.91 (1, 166)	*p* < 0.01	0.118
Erector Spinae + Multifidus	Group	18.72 (3, 166)	*p* < 0.01	0.253
	Age (years)	83.88 (1, 166)	*p* < 0.01	0.336
	Sex	64.42 (1, 166)	*p* < 0.01	0.280

Table 3 presents muscle‑specific median (IQR) fat‑fraction reference values grouped by physical‑activity level (sedentary vs. active) and by sex. Supplementary Fig. 4 visualises the FF distributions for these groups, panel A for activity group and panel B for sex.

**Table 3:** Median (IQR) intramuscular FF in percentage by physical activity group and by sex

Muscle	Sedentary (*n* = 87)	Active (*n* = 86)	Females (*n* = 92)	Males (*n* = 81)
Psoas	17.5 (16.0–19.1)	13.9 (12.8–15.2)	15.7 (13.3–17.9)	15.5 (13.7–17.0)
Iliacus	13.7 (11.9–15.3)	10.2 ( 9.4–11.3)	11.9 (10.1–14.1)	11.3 ( 10.0–13.4)
Quadratus Lumborum	16.2 (13.8–17.9)	13.2 (12.2–15.0)	15.0 (12.7–17.7)	14.0 (12.7–16.2)
Erector Spinae + Multifidus	25.7 (19.7–29.6)	19.2 (17.4–22.8)	25.6 (19.4–29.8)	19.0 (17.2–23.3)

## Discussion

This study demonstrates that a lightweight 3D U‑Net, trained with only 26 manual labels augmented by anatomically realistic deformations, can segment four lumbar spine muscles on Dixon MRI with high accuracy and automatically quantify FF, a measure of IMF, with manual level precision. The network not only outperformed a multi‑atlas baseline in all segmentation metrics but also allowed us to produce muscle specific reference FF ranges in a large healthy cohort, revealing strong effects of age and physical activity.

### Segmentation performance

Our results show that the 3D U-Net achieves high Dice similarity coefficients across all lumbar spine muscle groups, with the best performance for the P and ES + M muscles (Dices of 0.928 and 0.936 respectively) and slightly lower performance for the QL (Dice = 0.862). The relative volume difference (RVD) and Hausdorff distance metrics also confirm the robustness of our method. These results align with prior studies in musculoskeletal segmentation, which have reported that deep learning approaches outperform traditional methods in accuracy and reproducibility [14, 16, 36, 50]. The slight performance decrease for the QL could be attributed to its irregular shape, and its relatively smaller size, making accurate segmentation more challenging. Another region where segmentations were prone to errors was where P and I join near their insertion, affecting mainly I dice scores. This region is particularly difficult to segment even for experts. In the electronic supplementary material, segmentations with and without this issue are available.

Critical to this success was the nonlinear, muscle aware augmentation, which injected realistic anatomical variability and mitigated over fitting, a common issue when public lumbar muscle datasets are unavailable. This is particularly important in musculoskeletal MRI, where subject-specific anatomical differences can significantly affect segmentation performance. Our model achieved similar or better performance that other deep learning methods with larger manually segmented datasets [14, 36]. Our approach demonstrates that, even with a limited dataset, a deep learning method can be successfully trained by implementing advanced, domain-specific, augmentation techniques.

### Comparison with multi-atlas segmentation

The multi‑atlas approach, a popular choice when labels are scarce, struggled with inter‑subject differences and high IMF cases. Our 3D U‑Net delivered markedly higher Dice scores and lower HD while reducing inference time from minutes to seconds, supporting real‑time use in large‑scale studies or clinical workflows.

### Fat fraction measure accuracy and clinical relevance

Accurate segmentation is only valuable if biomarkers, such as FF, are equally reliable. Mean FF within a muscle mask quantifies IMF, which is key biomarker for muscle health, sarcopenia, and spinal pathology [7, 21, 51–54]. Automated FF correlated tightly with manual measurements (validation R² = 0.96, bias ≈ 0%), with 95% limits of agreement within ± 2.4%. No muscle‑specific systematic errors emerged, even for outlier cases with complete fatty replacement of QL, underscoring the pipeline’s robustness.

Reliable, fully automated IMF quantification makes longitudinal and large-scale assessments feasible. It offers an objective way to track age-related decline, disease progression, and responses to rehabilitation or pharmacological interventions, all settings in which small changes in FF can be clinically meaningful.

### Fat fraction reference values and determinants in healthy adults

The deep‑learning pipeline captured all inter‑muscle and inter‑subject differences with the same reliability as manual segmentation. In the 173 subject cohort, physiological heterogeneity among lumbar muscles was also confirmed. The ES + M complex showed the highest baseline FF and iliacus the lowest, mirroring patterns reported for gluteal muscles [52]. Multivariable analysis identified age and a sedentary lifestyle as the strongest predictors of elevated FF. Sex added a moderate contribution, and BMI was least influential.

These reference values provide new 3D normative data from a large cohort of healthy adults, contributing to the growing body of literature on lumbar muscle composition [55, 56], and extending available 2D CSA-based values reported in previous studies [57, 58].

When comparing the ES + M reference values to prior studies, it is important to consider differences in segmentation conventions. Specifically, there is ambiguity in the inclusion of posterior epimuscular fat tissue located between the epimyseal border and the lumbosacral fascia. In our study, we excluded this tissue based on anatomical considerations that define it as external to the erector spinae and multifidus compartments [40]. However, some published studies include this region in their segmentation of the paraspinal muscles, which may lead to higher reported FF values [41, 42].

Because these conclusions were drawn from a large cohort spanning a wide activity spectrum, these muscle‑specific reference distributions fill a gap in the literature and can aid future sarcopenia or spine‑degeneration studies.

### Clinical and research implications

Fully automated, reproducible IMF quantification enables longitudinal monitoring of spinal muscle health without the labour and variability inherent to manual labelling. Potential applications include early sarcopenia screening, risk stratification before spine surgery, and objective evaluation of exercise or pharmacologic interventions, where small FF changes are clinically meaningful.

### Limitations

While our deep learning model demonstrates quantitative results, several limitations should be noted. First, the training dataset comprises only 26 manually labelled scans of healthy individuals, although diverse, includes only healthy individuals and remains relatively small compared to large-scale imaging repositories (which are not available for muscle imaging). While nonlinear augmentation broadened anatomical diversity, a substantially larger set that includes pathological cases will be essential for full generalisability. Second, all images were acquired with an identical Dixon protocol on a single 3T scanner. Therefore, external validation across vendors, field strengths, resolutions and pulse‑sequence variants is a key next step and a major challenge in quantitative muscle MRI, where multicentre datasets are scarce and Dixon implementations vary substantially across platforms. Third, although our goal was not to introduce a new deep learning architecture or method, other newer methods than a 3D U-Net could potentially achieve even better performance. Lastly, our segmentation approach excluded the posterior epimuscular fat located between the epimyseal border and the lumbosacral fascia. While this choice aligns with prior anatomical definitions, it may limit direct comparison with studies that include this tissue in paraspinal muscle measurements.

## Conclusion

This study demonstrates that a 3D U-Net model, trained on just 26 manual labels and enhanced by anatomically realistic, nonlinear data augmentation, can accurately segment lumbar‑spine muscles and quantify muscle-specific FF from Dixon MRI. Applying the network on 173 asymptomatic adults spanning sedentary to highly active lifestyles, we generated novel normative FF reference ranges for these muscles in adults (ages 20–70) of both sexes. By reducing reliance on manual segmentation, this method enables large-scale investigations of sarcopenia, spine disorders, and muscle degeneration, with valuable applications in both research and clinical contexts.

## Supplementary Information

Below is the link to the electronic supplementary material.

Supplementary Material 1

Supplementary Material 2

## Data Availability

Part of the data is available from the corresponding author on reasonable request.
