# Supplementary material for: Automated deep-learning quantification of intramuscular fat in lumbar spine muscles on Dixon MRI: validation and normative reference values from 173 healthy adults
Source: BMC Med Imaging. 2026 Mar 31;26:220. doi: 10.1186/s12880-026-02329-9 (PMC13130419; doi:10.1186/s12880-026-02329-9)
Supplement: Supplementary file 1 — Supplementary Material 1 [file 12880_2026_2329_MOESM1_ESM.docx]

**Supplementary Material for “Automated Deep-Learning Quantification of Intramuscular Fat in Lumbar Spine Muscles on Dixon MRI: Validation and Normative Reference Values from 173 Healthy Adults”**

**Supplementary Figure 1 – Exclusion of Epimuscular Fat**


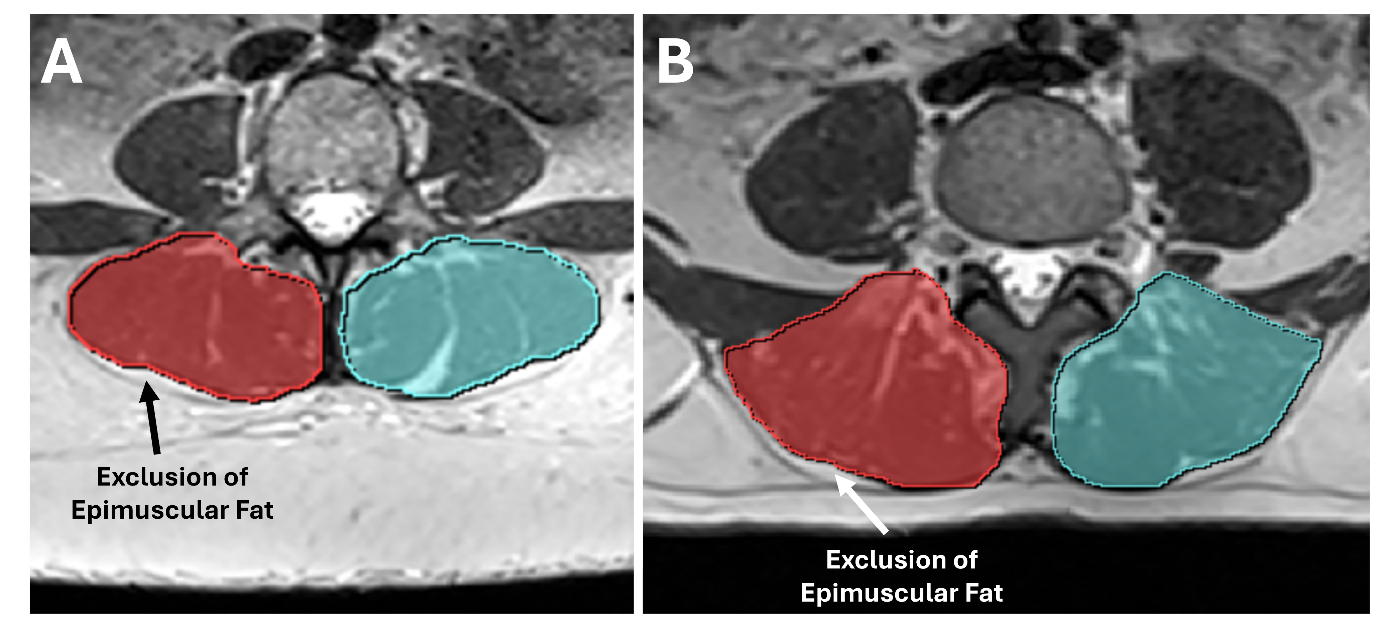
Supplementary Figure 1. Two examples of the posterior delineation of the paraspinal ES+M complex, where the epimyseal border is used to define the posterior boundary. The epimuscular fat tent, excluded from the segmentation, is indicated by arrows in both panels.

**Supplementary Figure 2 – Example of Nonlinear Data Augmentation**


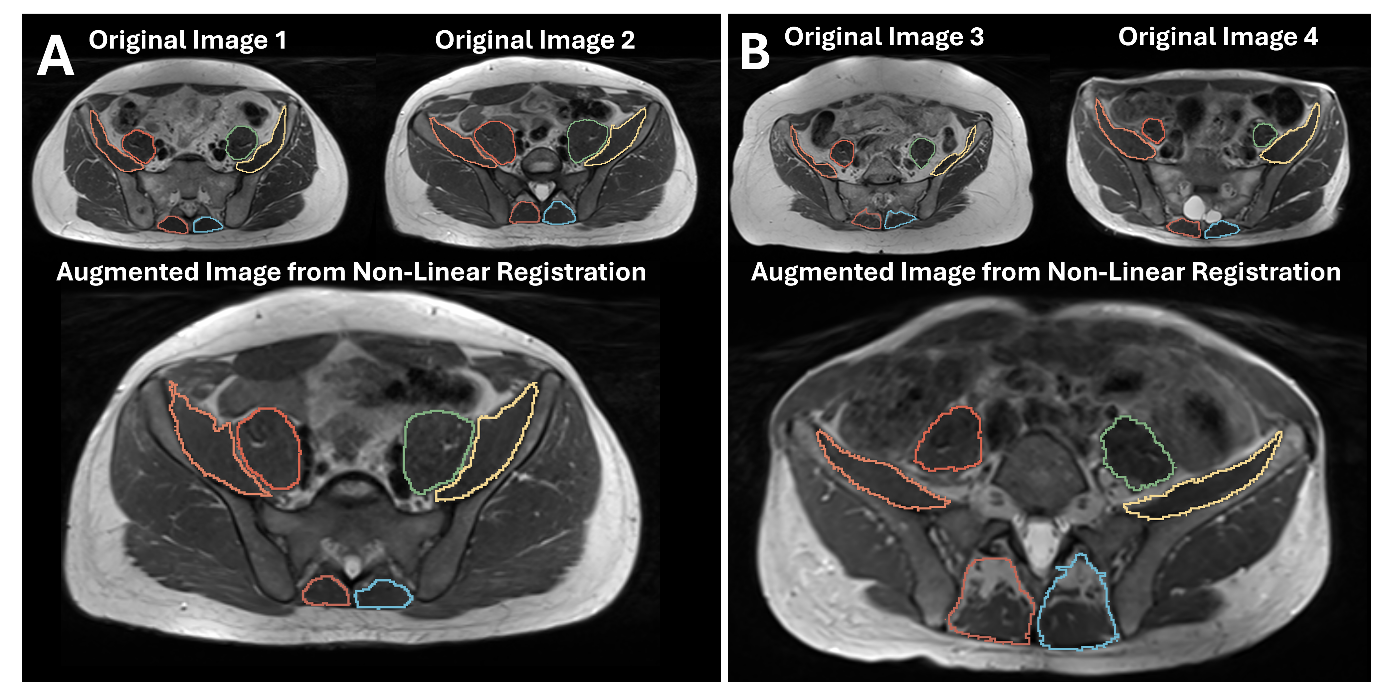
Supplementary Figure 2. Example of two augmented images using nonlinear registration between two manually segmented images. Top: original images involved in the pairwise B-spline registration. Bottom: an augmented image resulting from an early stopped registration.

**Supplementary Figure 3 – Bland-Altman Analysis of Automated FF vs Manual**


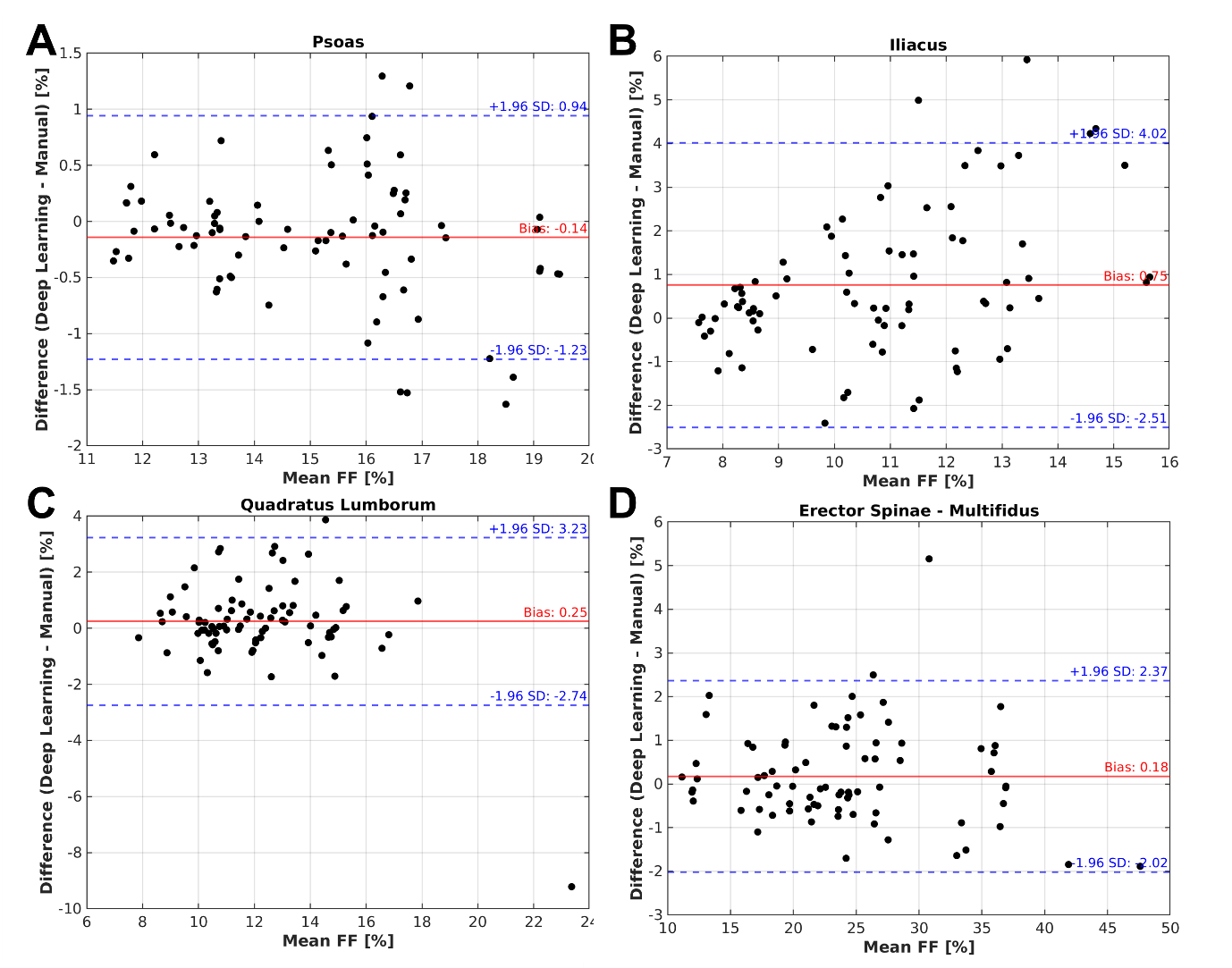
Supplementary Figure 3. Bland-Altman analysis of the FF for psoas (A), iliacus (B), quadratus lumborum (C) and erector spinae - multifidus (D), comparing deep learning and manual segmentations. The solid lines represent mean differences (bias), and dashed lines indicate limits of agreement (±1.96 standard deviations). Each plot shows the mean FF of the two methods on the x-axis against their differences on the y-axis, illustrating consistency and variability across methods.

**Supplementary Figure 4 – FF distributions by sex and physical activity groups**


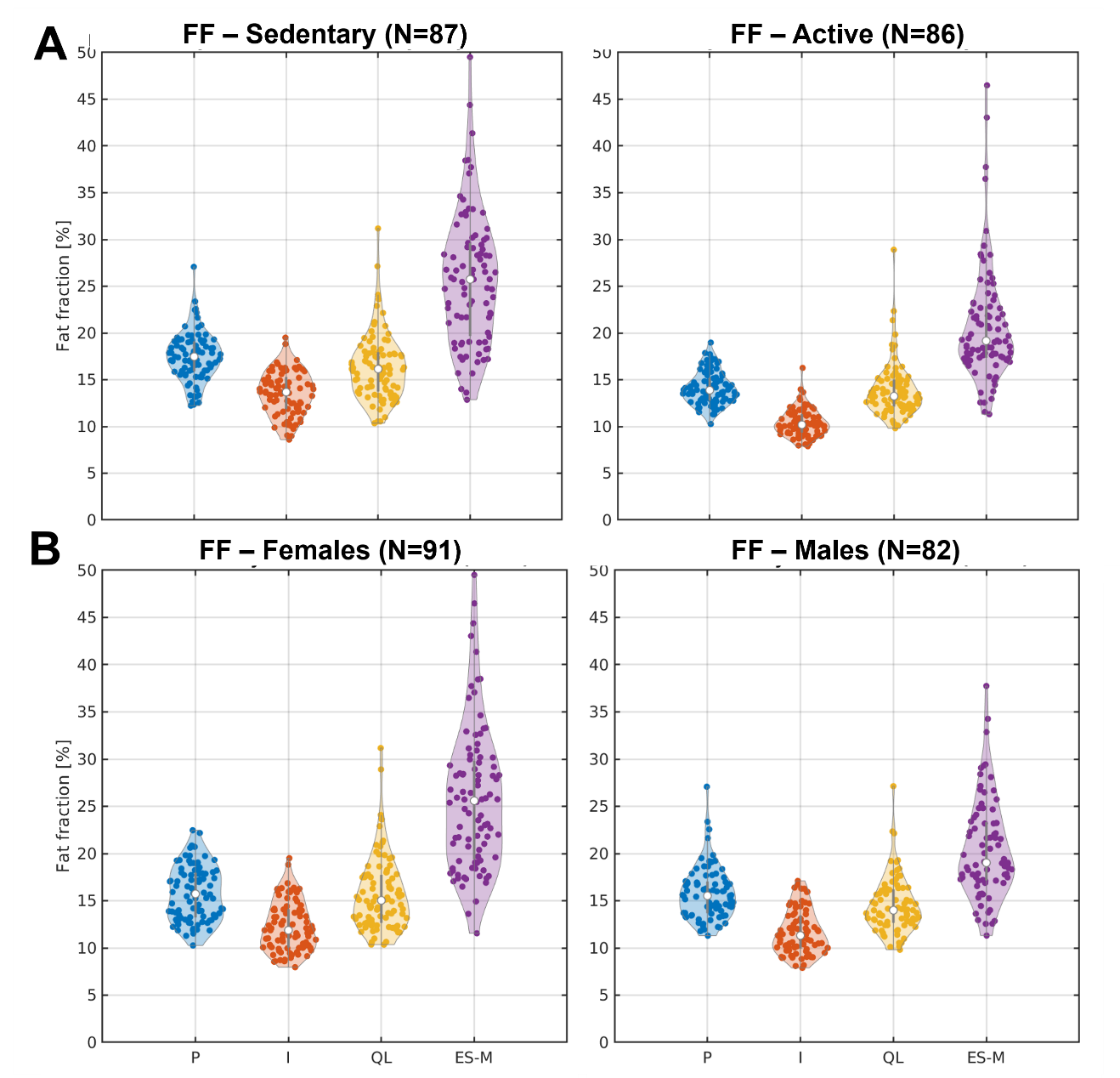


Supplementary Figure 4. Violin plots illustrating the distribution of FF values for the full dataset grouped by physical activity (A) and sex(B), measured with the deep learning-based automated method.

**Digital Supplementary Material**

In addition, four animated gifs showing the segmentation accuracy have been uploaded. Each gif goes through each axial slice showing the outline of the automated and manual segmentations.
